# Selenium, zinc and magnesium: serum levels in members of the Czech Republic rescue fire brigade

**DOI:** 10.2478/v10102-010-0055-6

**Published:** 2010-12

**Authors:** Hana Střítecká, Pavol Hlubik

**Affiliations:** University of Defence, Faculty of Military Health Science, Department of Military Hygiene, Hradec Kralove, Czech Republic

**Keywords:** selenium, zinc, magnesium, serum concentration, cardiovascular disease

## Abstract

The Czech Republic ranks among the countries with the highest prevalence of dyslipoproteinemia and cardiovascular diseases (CVD). As a valid tool in their primary prevention, the authors consider monitoring of selected components (including metal ions and vitamins) of the body′s antioxidant system.

The study was focused on monitoring the health condition of members of the Czech Republic Rescue Fire Brigade. The concept of the study made it possible to reveal relationships between the serum magnesium, zinc, selenium levels and the age or biochemical and anthropometrical parameters generally used as risk indices of cardiovascular disease. The results contribute to the information about normal values of serum magnesium, zinc and selenium concentrations in the Czech population. The mean serum concentration of magnesium was 0.82 ± 0.06 mmol/l, that of zinc was 18.25 ± 2.54 µmol/l, and the mean selenium serum concentration was 0.80 ± 0.14 µmol/l.

## Introduction

Sufficient intake of essential substances is a condition for satisfactory nutrition. Deficiency of one of them can lead to serious health damage; it can even endanger life. The need of individual nutrients for the human organism has been verified under ever new connections in many metabolic studies in people, in experimental work on animals, and also in clinical and epidemiological monitoring. This need has been quantified in the final phase in the form of reference intake values of individual nutrients, which are the basis for quality assessment of our food and our nutrition (Garrow *et al*., 2000).

Many trace elements, although present in minute quantities in man, are essential nutrients. Their presence was long overlooked and it has only been in recent years that analytical techniques capable of measuring such trace levels have been developed. These elements perform functions indispensable for the maintenance of life, growth and reproduction. Numerous enzymes require a small and constant number of atoms of metal per mole to attain full activity. Minute variations can impair or substantially increase the activity of these enzymes while removal of the metal by dialysis against a chelating agent may decrease the activity to zero (Agay *et al*., 2005).

The trace elements, *e.g.* zinc, selenium, magnesium, though present in very small amounts, perform highly specialized functions in initiating many biological reactions and they play important roles in the biological behavior of cells. Thus their altered levels may be either favorable or detrimental in health and disease (Bank *et al*., 2007).

Selenium is a trace mineral that is essential to good health but it is required only in small amounts Selenium is incorporated into proteins to make selenoproteins, which are important antioxidant enzymes. The antioxidant properties of selenoproteins help prevent cellular damage from free radicals that may contribute to the development of chronic diseases such as cancer and heart disease. Other selenoproteins help regulate thyroid function and play a role in the immune system (Thomson, 2004; Goldhaber, 2003).

Magnesium is the fourth most abundant mineral in the body and is essential to good health. Approximately 50% of total body magnesium is found in bone. The other half is found predominantly inside cells of body tissues and organs. Only 1% of magnesium is found in blood, but the body works very hard to keep blood levels of magnesium constant. Magnesium is needed for more than 300 biochemical reactions and is associated with a large number of enzymes in the body. It helps maintain normal muscle and nerve function, keeps heart rhythm steady, supports a healthy immune system, and keeps bones strong. Magnesium also helps regulate blood sugar levels, promotes normal blood pressure, and is known to be involved in energy metabolism and protein synthesis. There is an increased interest in the role of magnesium in preventing and managing disorders such as hypertension, cardiovascular disease, and diabetes (Saris *et al*., 2000; USDA, 2003).

Zinc is an essential mineral that is naturally present in some foods, added to others, and available as a dietary supplement. Zinc is involved in numerous aspects of cellular metabolism. It is required for the catalytic activity of approximately 100 enzymes and it plays a role in immune function, protein synthesis, wound healing, DNA synthesis, and cell division. Zinc also supports normal growth and development during pregnancy, childhood, and adolescence and is required for proper sense of taste and smell (Trumbo *et al*., 2001; Maret & Sandstead, 2006).

## Methods

The project involved 933 healthy volunteers (only males) of the Czech Republic Rescue Fire Brigades from selected areas of the Czech Republic (Praha, Beroun, Klatovy, Kroměříž, Nový Jičín a Ústí nad Labem). The average age of the group was 35.2 ± 8.1 years. Physical activity outside their job was assessed: 41.6% of the study participants stated that they exercised regularly, 54.2% irregularly and 4.2% not at all. Non-smokers constituted 82% of all volunteers and 18% of the volunteers stated that they smoked.

Venous blood was taken after overnight fast to determine biochemical parameters. Anthropometrical examination include weight, height, caliperation of waist and hip circumference. In order to assess the actual health condition of the volunteers and to record their eating habits, all study participants received simple questionnaires, which were focused on the consumption of meat, fish, milk, eggs, vegetables, fruit, alcohol and supplements with vitamin preparations.

Blood for plasma analyses was drawn into heparin tubes. The blood was centrifuged at room temperature for 10 minutes at 3 000rpm; plasma was pipetted into plastic vials and kept at –20°C until analysis. The serum selenium, magnesium and zinc concentrations were determined by direct electrothermal atomic – absorption spectrometry on AAS Unicam, GB. Serum mineralized in microwave system (Milestone, Italy) provided selenium analysis. In sample preparation, mineralization in a microwave digestion system was used.

## Results

**Figure 1:**
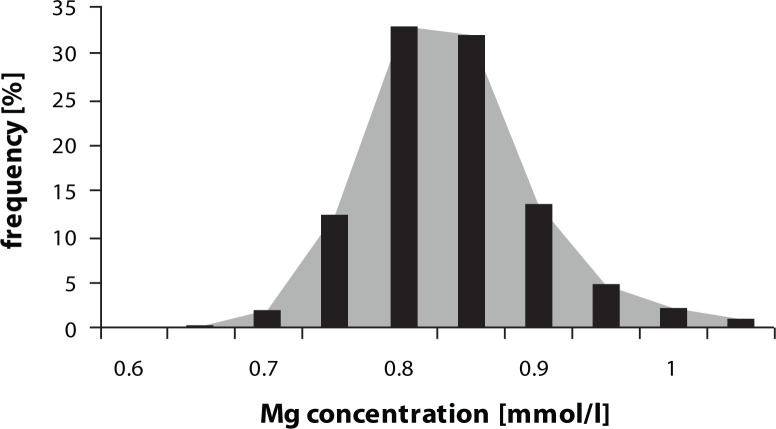
Distribution of serum Mg concentration.

**Figure 2:**
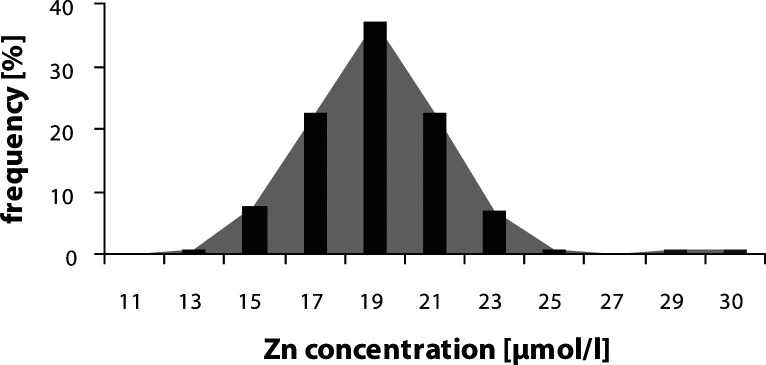
Distribution of serum Zn concentration.

**Figure 3:**
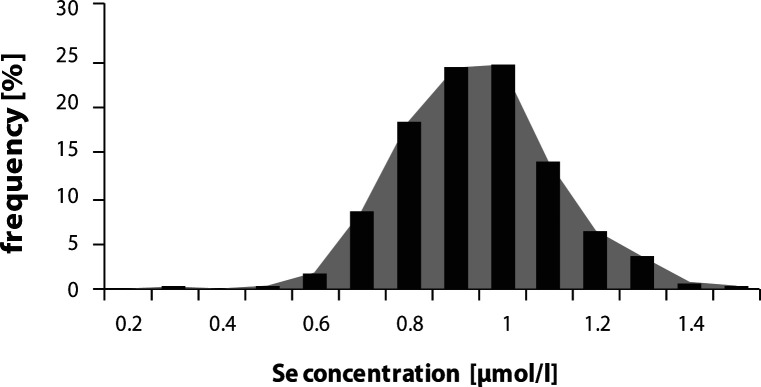
Distribution of serum Se concentration

The mean serum concentration of magnesium was 0.82 ± 0.06 mmol/l, of zinc 18.25 ± 2.54 µmol/l and the mean selenium serum concentration was 0.80 ± 0.14 µmol/l. The distribution of magnesium (Mg) (Figure 1), zinc (Zn) (Figure 2) and selenium (Se) (Figure 3) serum concentrations in the population group examined approached normal values.

## Discussion

In the group monitored, the average serum concentrations of magnesium and selenium showed a tendency towards lower values of the so-called physiological range, however, they corresponded with other findings within the Czech population (Table 1). The concentrations of zinc corresponded with commonly found normal values (Zima, 2002).

**Table 1:** Distribution of serum concentration value in the Czech population.

					Percentiles			
					
	mean	sd	median	Q3-Q1/2	5^th^	10^th^	90^th^	95^th^
**Mg** [mmol/l]	0.82	0.06	0.81	0.04	0.72	0.74	0.88	0.92
**Zn** [µmol/l]	18.25	2.54	18.00	1.56	14.74	15.25	21.00	21.75
**Se** [µmol/l]	0.80	0.14	0.80	0.11	0.56	0.60	1.01	1.10

The mean selenium serum concentration was 0.80 ± 0.14 µmol/l. Strong geographic variations in selenium content of food supplies were observed, with very low nutritional Se intake in the Czech population. The results of serum selenium determination confirmed a mild to severe selenium deficiency in the population in northeastern Bohemia (Table 2) (Kvíčala *et al*., 1999; Beneš *et al*., 2000).

**Table 2:** Evaluation of serum selenium concentrations in inhabitants of CZ and the monitored group.

Evaluation	Se in serum (mg/l).Range	Frequency (%) Inhabitants of CZ	Frequency (%) Monitored group
**Pharmacol. level**	>140	0.1	6.6
**Optimal level**	100–140	0.7	20.0
**Marginal deficiency**	70–100	8.6	23.3
**Mild deficiency**	55–70	25.6	15.0
**Deficiency**	45–55	28.0	4.0
**Severe deficiency**	<45	37.0	31.1

The mean serum concentration of zinc was 18.25 ± 2.54 µmol/l. A satisfactory zinc status was found in the Czech population, with a mean serum zinc level of 13.90 ± 4.22 µmol/l. The frequency analysis proved however one-third of the inhabitants to have serum zinc concentrations below the cutoff value of 12.2 µmol/l (Beneš *et al*., 2000).

The mean serum concentration of magnesium was 0.82 ± 0.06 mmol/l. The serum magnesium concentrations in the Czech population were found to be in the range of 0.66–1.15 mmol/l (Zima, 2002; Šimečková *et al*., 1999).

**Figure 4:**
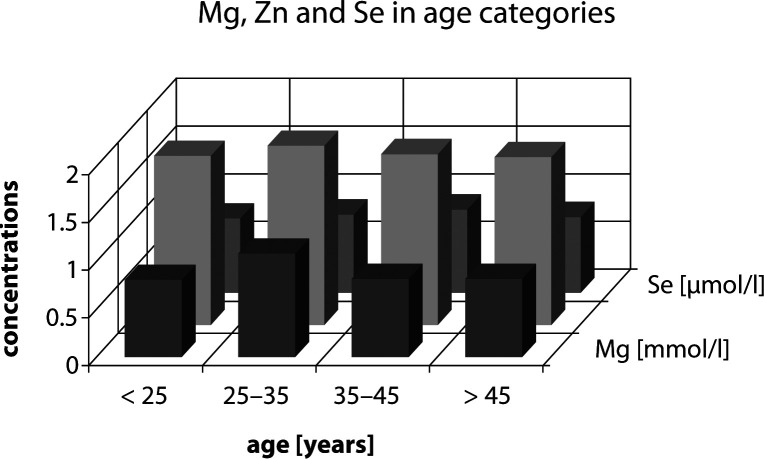
Distribution of serum Mg, Zn and Se concentration in age categories.

The serum magnesium, zinc and selenium levels in the subjects examined were classified in four age categories evaluated by analysis of variance. The highest mean values of magnesium and zinc serum concentrations were found in the category 25–35 years; the highest mean value of selenium serum concentration was calculated for the category 35–45 years. No statistically significant differences were found between magnesium, zinc and selenium serum concentrations in the four age categories (Figure 4).

The concept of the study made it possible to reveal relations between the serum magnesium, zinc and selenium levels and the age or biochemical and anthropometrical parameters which are generally used as risk indices of cardiovascular disease. The results of the statistical evaluation are documented in the form of a correlation matrix (Table 3). No statistically significant relations between the age of the subjects examined and their serum magnesium, zinc and selenium concentrations were proved (these findings were confirmed also by analysis of variance). No statistically significant correlations were found among magnesium serum concentrations and the other parameters followed. Statistically significant negative correlations were revealed for relations between zinc serum concentration and cholesterolemia – TCH (*p*≤0.05) and triacylglycerolemia – TAG (*p≤*0.01). Statistically significant (*p*≤0.01) negative correlation was proved among selenium serum concentration and ascorbemia (vitaminC).

**Table 3:** Correlation matrix between the serum magnesium, zinc and selenium levels and parameters which are generally used as risk indices of cardiovascular disease.

	Correlation matrix								
	
	age	BMI	waist	Mg	Zn	Se	TCH	TAG	vit. C
**age**	1.00**								
**BMI**	0.265**	1.00**							
**waist**	0.356**	0.844**	1.00**						
**Mg**	ns	ns	ns	1.00**					
**Zn**	ns	ns	ns	ns	1.00**				
**Se**	ns	ns	ns	ns	ns	1.00**			
**TCH**	0.446**	0.248*	0.282**	ns	–0.234*	ns	1.00**		
**TAG**	0.204*	0.343**	0.399**	ns	–0.317**	ns	0.419**	1.00**	
**vit. C**	–0.279**	ns	ns	ns	ns	–0.271**	–0.212*	ns	1.00**

Statistical significance of r:

* *p* ≤ 0.05;

** *p* ≤ 0.01

ns = no significance

**Figure 5:**
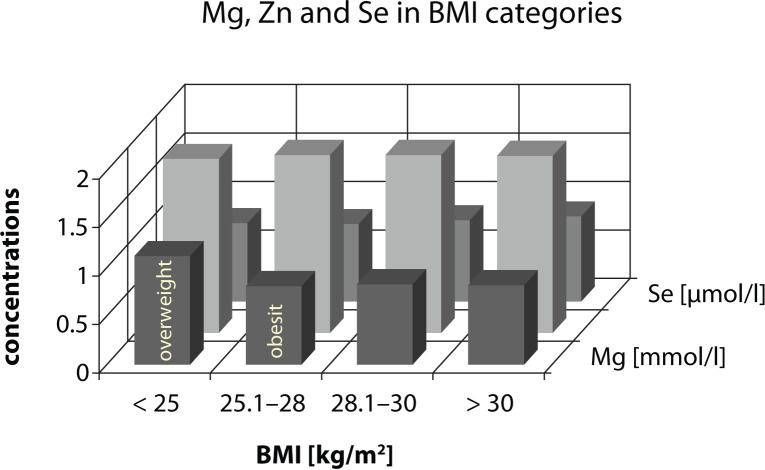
Distribution of serum Mg, Zn and Se concentration in BMI categories

The high incidence of obesity and overweight in the population group examined prompted the evaluation of magnesium, zinc and selenium saturation in subjects classified as 4 BMI (body mass index=ratio of body weight and square of body height) categories. The magnesium, zinc and selenium serum concentrations in subjects with normal BMI (20–25kg/m^2^) were compared with concentrations in the category of overweight 1^st^ grade (BMI 25.1–28.0kg/m^2^), overweight 2^nd^ grade (28.1–30.0kg/m^2^) and the category of obesity (BMI over 30kg/m^2^) by analysis of variance. Magnesium concentrations showed the tendency to decrease with higher BMI, while zinc and selenium serum concentrations showed a tendency to increase in higher BMI categories. No statistical significance of these changes was established (Figure 5, Table 4).

**Table 4:** Distribution of Mg, Zn and Se serum concentration in BMI categories.

	Serum concentrations in BMI categories			
	
	normal	1^st^ grade overw.	2^nd ^grade overw.	obesity
**Mg** [mmol/l]	1.12	0.81	0.83	0.82
**Zn** [µmol/l]	18.0	18.4	18.4	18.3
**Se** [µmol/l]	0.81	0.80	0.84	0.88

It is clear from the questionnaires that almost 20% of the volunteers examined used regularly preparations for antioxidant supplementation (predominantly supplementation of vitamin C and beta carotene, together wit the selenium and lutein). The effect of regular supplementation was only seen in not significantly increased vitaminC levels.

## Conclusion

The health state of each individual depends on many objective and subjective conditions. Among objective factors that influence man's health genetic preconditions and the influence of the working milieu and life environment can be ranked. Man's lifestyle plays an important role in influencing health.

In addition to the vital role that trace elements play in enzymatic reactions, they have been examined critically as a potential key factor in varied diseases, including cancer and cardiovascular disease. Although trace elements are only a part of the total picture, they are important in the relationship of nutrition and maintenance of health as well as in prevention of disease.

The serum level of the elements monitored in the selected group corresponded with findings within the Czech population.
